# The Global Need for Easy and Valid Assessment Tools for Orofacial Pain

**DOI:** 10.1177/00220345221110443

**Published:** 2022-07-26

**Authors:** F. Lobbezoo, G. Aarab, F.P. Kapos, A.F. Dayo, Z. Huang, M. Koutris, M.A. Peres, M. Thymi, B. Häggman-Henrikson

**Affiliations:** 1 Department of Orofacial Pain and Dysfunction, Academic Centre for Dentistry Amsterdam, University of Amsterdam and Vrije Universiteit Amsterdam, Amsterdam, the Netherlands; 2 Center for Child Health, Behavior and Development, Seattle Children’s Research Institute, Seattle, WA, United States; 3 Department of Oral Medicine, School of Dental Medicine, University of Pennsylvania, Philadelphia, PA, USA; 4 National Dental Research Institute Singapore, National Dental Centre Singapore, Singapore; 5 Oral Health ACP, Health Services and Systems Research Programme, Duke-NUS Medical School, Singapore; 6 Department of Orofacial Pain and Jaw Function, Malmö University, Malmö, Sweden

**Keywords:** facial pain, temporomandibular joint disorders, patient outcome assessment, reproducibility of results, global health, health equity

## Abstract

The World Health Organization recently adopted a historic resolution (WHA74.5) on the urgent need for global oral health improvement. This resolution is particularly relevant in the perspective of the high prevalence of untreated oral diseases. However, one important aspect has been mentioned only in passing, namely that poor oral health often leads to orofacial pain, which is the most common reason for emergency dental visits worldwide. Therefore, an evidence-based decision-making process on oral health should include data related to orofacial pain complaints. To that end, the availability of reliable and valid assessment tools of orofacial pain and related treatment outcomes is essential. INfORM (International Network for Orofacial Pain and Related Disorders Methodology) of the International Association for Dental Research has been one of the driving forces behind the development and implementation of comprehensive sets of tools for such assessments. However, as a prerequisite for the desired global implementation, reliable and valid tools that are also brief, easy to translate, and culturally adaptable need to be further developed and tested. Some of the groundwork to facilitate this process has already been carried out. In addition, a working group within INfORM has developed a short clinical assessment tool for orofacial pain diagnostics that is near completion and will soon be ready for dissemination. Ultimately, reliable and valid orofacial pain assessment is a necessary step toward the development and implementation of appropriate “best buy” interventions that address this major driver of need for oral health care worldwide.

## Urgent Need for Global Oral Health Improvement

In May 2021, the 74th World Health Assembly of the World Health Organization (WHO; 2021) adopted a historic resolution, WHA74.5, on the urgent need for global improvement of oral health. This resolution reinforces the recognition by the WHO (2022a) that “oral health is a key indicator of overall health, well-being and quality of life.” The seriousness of the new resolution resides in the high prevalence of untreated oral conditions, most notably dental caries (tooth decay), severe periodontal (gum) disease, and extensive tooth loss, affecting around 3.5 billion individuals worldwide (Peres et al. 2019). Poor oral health is not only associated with reduced quality of life, disruption to family life, lost school days, and decreased work productivity (Peres et al. 2019) but has also been suggested to be associated with general health conditions such as cardiovascular disease, diabetes, cancer, pneumonia, premature birth, obesity, obstructive sleep apnea, and dementia (Suzuki et al. 2016; Seitz et al. 2019; Nadim et al. 2020). The ensuing economic burden for our society is enormous: the direct and indirect costs associated with oral health problems have been estimated at US $545 billion per annum worldwide, which is comparable to the economic burden of the 2 most expensive illnesses: cardiovascular diseases and diabetes (Righolt et al. 2018).

## Oral Health and Orofacial Pain

We would, however, like to draw attention to an important aspect that has been mentioned in passing but not elaborated on, neither in the WHA74.5 resolution (WHO 2021) nor in the recently published global strategy on oral health (WHO 2022b)— namely, that poor oral health, among other patient-reported complaints, commonly leads to pain in the orofacial area. The importance of being orofacial pain free as minimum requirement for good oral health has been recognized by the FDI World Dental Federation (2022), which defines oral health as “multi-faceted and includes the ability to speak, smile, smell, taste, touch, chew, swallow and convey a range of emotions through facial expressions with confidence and without pain, discomfort and disease of the craniofacial complex (head, face, and oral cavity).” While dental caries and periodontal diseases have traditionally been logical targets for oral health care and prevention strategies, a study with dentists from all WHO regions found orofacial pain to be the most common reason for emergency dental visits worldwide (John et al. 2020).

Acute orofacial pain is most often odontogenic (Lipton et al. 1993) and, in a majority of cases, directly related to oral health and untreated dental conditions such as tooth decay. This is especially relevant in low- and middle-income countries, where access to emergency dental care is limited. Hence, complaints of orofacial pain ensuing from oral diseases, as well as primary orofacial pain disorders (i.e., where pain is not a symptom of another underlying disease, but the pain itself is the main feature of the condition), are driving patients to visit oral health care professionals. More than 10% of the general adult population report pain in the orofacial region (Lipton et al. 1993; Häggman-Henrikson et al. 2020). However, these data refer to high-income countries, while population estimates are not available from many low- and middle-income countries. Therefore, to enable the adequate assessment and evaluation of the presence of orofacial pain in low- and middle-income countries as well as in disadvantaged communities, new strategies are needed.

As mentioned earlier, acute orofacial pain is often related to the teeth (Lipton et al. 1993), whereas chronic orofacial pain can be related to a number of causes ranging from neuropathic pain to pain conditions with a musculoskeletal origin—specifically, temporomandibular disorders (TMDs; Maixner et al. 2011). TMD is the most common cause for chronic orofacial pain and is an umbrella term that embodies pain and dysfunction involving the masticatory muscles, the temporomandibular joints, and associated structures (Lipton et al. 1993). Chronic orofacial pain, like other chronic pain conditions (Blyth and Huckel Schneider 2018), has considerable impact on the quality of life of the individual, with substantial societal costs (Durham et al. 2016).

Over the past decades, the assessment of TMDs has been the focus of many international initiatives. Ohrbach and Dworkin (2016) described the many steps taken toward finalization of the Diagnostic Criteria for Temporomandibular Disorders (DC/TMD; Schiffman et al. 2014) and the Expanded Taxonomy of the DC/TMD (Peck et al. 2014). Together, these systems allow the structured assessment of common and less common TMDs. The International Network for Orofacial Pain and Related Disorders Methodology (INfORM) of the International Association for Dental Research (IADR; 2022) has been one of the driving forces behind the development and implementation of the DC/TMD and the Expanded Taxonomy. In addition, INfORM has been an important partner in the development of the International Classification of Orofacial Pain (ICOP; 2020), with the Orofacial and Head Pain Special Interest Group of the International Association for the Study of Pain, the American Academy of Orofacial Pain, and the International Headache Society. However, even though INfORM represents an international network of orofacial pain researchers and clinicians, it does not yet manage to reach all countries and communities that might benefit from the network’s tools. People with orofacial pain in low- and middle-income countries are less likely to receive care using comprehensive diagnostic tools such as the DC/TMD; barriers include lack of local language translations and representation in the international knowledge exchange in the field of orofacial pain (Figure). Therefore, during a recent workshop organized by INfORM during the 2021 General Session of the IADR, it was concluded that one of the future goals should be to 1) develop assessment tools for TMDs and other orofacial pain conditions that are easy to use yet reliable and valid, 2) enable global dissemination and implementation, and 3) enhance access by low- and middle-income countries as well as disadvantaged communities. In parallel, outreach activities would seek to broaden INfORM’s engagement with local partners, especially in underserved areas, thus maximizing the relevance and applicability of its work.

**Figure.**
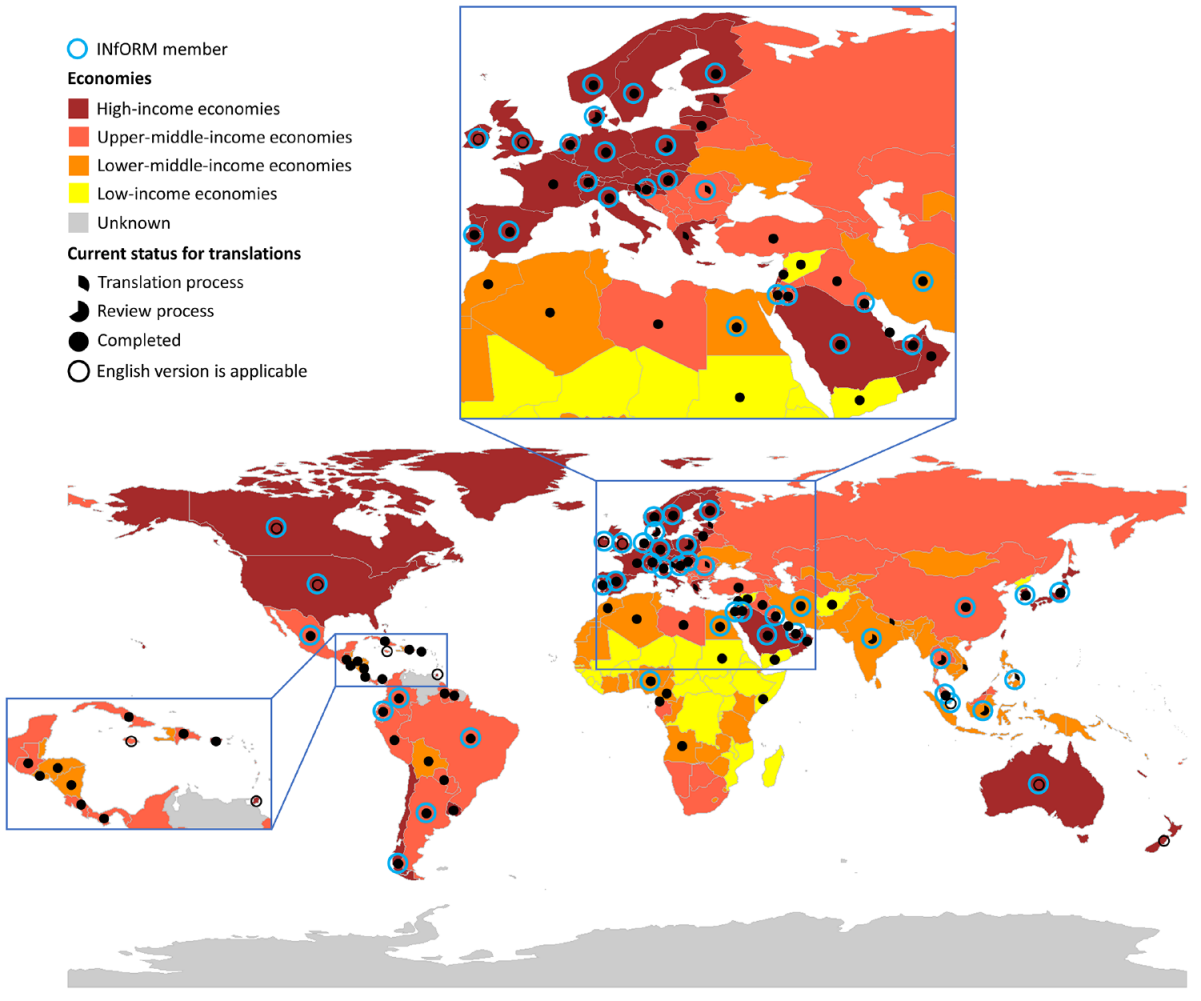
Global map shows the world’s countries by economy: low, lower-middle, upper-middle, and high income. The full and partial black dots and black circles represent the availability of local versions of the DC/TMD (Diagnostic Criteria for Temporomandibular Disorders): full black dots represent finalized translated and culturally adapted local versions; partial dots, local versions that are currently being translated or reviewed; and black circles, countries where the source version (English language) can be applied. The blue circles indicate countries with ≥1 members of INfORM (International Network for Orofacial Pain and Related Disorders Methodology). Clearly, the lower the income level, the less likely that a local version of the DC/TMD is available.

In what follows, this future goal is described in relation to the urgent need for global oral health improvement as initiated and promoted by the WHO. Furthermore, current initiatives are outlined toward the development and implementation of easy-to-use yet psychometrically sound tools for the assessment of orofacial pain conditions, along with future directions.

## Global Initiatives

In the WHA74.5 resolution (WHO 2021), an ambitious request is formulated to develop a global oral health strategy by 2022 (WHO 2022b) and to translate this strategy into a comprehensive global action plan for public oral health by 2023, with specific, measurable, and realistic targets to be achieved by 2030. Importantly, the resolution states that this action plan should provide “a basis for a healthy mouth, where no one is left behind” (WHO 2021). Unfortunately, the legacy of historical inequities as well as ongoing policies and practices worldwide place much of the burden of untreated oral diseases on socially marginalized and economically disadvantaged communities where oral health care and prevention are largely inaccessible (Peres et al. 2019). To address this, the recently established Lancet Commission on Oral Health has formulated 6 key recommendations to guide the new WHO global oral health strategy: 1) include diverse voices and engage communities, 2) place equity and social justice at the core, 3) tackle major risk factors such as sugar intake, 4) embrace major system reforms, 5) create better data for decision making, and 6) close financing gaps (Benzian et al. 2021). In addition, Lobbezoo and Aarab (2021) suggested the following seventh key recommendation: 7) promote interprofessional collaboration between medical doctors and dentists in research, education, prevention, and care provision. It is the commission’s hope and expectation that when the WHO, its member states, and their partners address these issues, a tangible improvement of global oral health will be achieved, in line with the ambitions of the WHA74.5 resolution (WHO 2021) and the recently published global strategy on oral health (WHO 2022b).

The Global Burden of Diseases (GBD; 2019) study assesses mortality and disability for >300 diseases and injuries, including untreated dental caries in the permanent and primary dentition, severe periodontitis, complete edentulism, extensive tooth loss, and oral cancer. However, other possible causes of orofacial pain, such as TMDs, are not explicitly addressed. Orofacial pain disorders might have fit under “other oral disorders” or “other musculoskeletal disorders”; however, the reported prevalence of these broad “other” disorder categories in the GBD (2019) study—1.8% and 5.5%, respectively—suggests a massive underestimation of the global burden of orofacial pain (e.g., 3.4% of the general population was estimated to have frequent pain in the temple, face, jaw, or jaw joint; Lövgren et al. 2016). The lack of widespread global accessibility to assessment tools for orofacial pain that are easy to use yet valid likely contributes to this gap. Although oral disorders as a whole have been found in the top 10 ranked causes of disability between 1990 and 2019 worldwide (GBD 2019), their importance would be recognized as even greater if orofacial pain disorders were adequately captured.

## Tools for Screening and Assessment of Orofacial Pain

The usage of instruments for the screening and assessment of orofacial pain conditions should be rooted in the biopsychosocial model, thereby reflecting the fact that such conditions are not only related to biological factors but also highly dependent on psychological and social factors (Gatchel et al. 2007; Ohrbach and Dworkin 2016), with far-reaching psychosocial consequences. INfORM has been instrumental in promoting the translation, dissemination, and implementation of accurate and appropriate tools for the assessment and evaluation of various types of orofacial pain conditions, especially TMDs, in a biopsychosocial framework. Many tools are available in multiple languages from the INfORM (2022) website. Diagnostic systems such as the DC/TMD (Schiffman 2014), the Expanded Taxonomy (Peck et al. 2014), and the ICOP (2020) are comprehensive and have greatly contributed to advancement of the field, albeit in mainly high-income countries so far (Figure).

The availability of easy-to-use, reliable, valid, and feasible tools for the assessment and evaluation of orofacial pain conditions is a prerequisite for a truly global implementation involving low- and middle-income countries. This means that such instruments need to be developed, tested, disseminated, and implemented. Apart from having good psychometric properties in terms of internal consistency, reliability, and validity, these tools need to be brief, easy to translate, culturally adaptable, and above all suitable for global dissemination and implementation so that, as the WHA74.5 resolution states, “no one is left behind” (WHO 2021). Given the pressing nature of this resolution, it is crucial that the development and implementation of such brief tools in relation to orofacial pain conditions be dealt with urgently. Fortunately, some of the groundwork to facilitate this process has already been covered. For example, different brief screening tools have been developed and proposed for dissemination and implementation for orofacial pain and TMDs (Gonzalez et al. 2011; Zhao et al. 2011; Lövgren et al. 2016). The development of brief screening tools for other orofacial pain conditions is being planned as well, using the ICOP (2020) as a starting point. In addition, a working group within INfORM has developed a short version of the DC/TMD for the assessment of orofacial pain and TMDs that is near completion and will soon be ready for dissemination. The aim of this short version is to facilitate dissemination in general dentistry as well as in disadvantaged communities and low- and middle-income countries.

## Toward an Orofacial Pain-free World

On the basis of this elaboration, we are confident that this ongoing work can help to strengthen the WHO global oral health strategy by delivering and implementing suitable tools for the easy yet valid assessment of orofacial pain conditions worldwide within a short time frame. To that end, INfORM will seek collaboration with the Global Oral Health Inequalities Research Network of the IADR, which has excellent contacts in all WHO regions. Ultimately, the assessment of orofacial pain conditions will allow for the development and implementation of appropriate “best buy” interventions (WHO 2021) that address this major driver of the need for oral health care for all. As such, work already carried out and in progress by organizations such as INfORM and the Global Oral Health Inequalities Research Network can make a crucial contribution to an orofacial pain–free world.

## Author Contributions

F. Lobbezoo, G. Aarab, B. Häggman-Henrikson, contributed to conception, drafted and critically revised the manuscript; F.P. Kapos, A.F. Dayo, Z. Huang, M. Koutris, M.A. Peres, M. Thymi, contributed to conception, critically revised the manuscript. All authors gave final approval and agree to be accountable for all aspects of the work.
